# Numerical Simulation of the Effect of Pulmonary Vascular Resistance on the Hemodynamics of Reoperation After Failure of One and a Half Ventricle Repair

**DOI:** 10.3389/fphys.2020.00207

**Published:** 2020-03-17

**Authors:** Yan Fu, Aike Qiao, Yao Yang, Xiangming Fan

**Affiliations:** ^1^College of Life Science and Bioengineering, Beijing University of Technology, Beijing, China; ^2^Beijing Anzhen Hospital, Capital Medical University, Beijing, China

**Keywords:** one and a half ventricle repair, pulmonary vascular resistance, lumped parameter model, hemodynamics, numerical simulation

## Abstract

**Objective:**

The one and a half ventricle repair (1.5VR) is a common clinical choice for patients with right heart dysfunction. Considering the influence of blood circulation failure and reoperation in urgent need, this essay aims to explore the hemodynamic effects of different pulmonary vascular resistance (PVR) values on reoperation after 1.5VR failure.

**Methods:**

The lumped parameter model (LPM) was used to simulate the reoperation, including the return biventricular repair (2VR), ligation of azygos vein (1.5VR′) and return single ventricular repair (1.0VR). Firstly, the debugging parameters were used to simulate the hemodynamics of 2VR. Secondly, the value of PVR was changed from one to four times while the other parameters remained unchanged. Finally, 15 cardiac cycles were simulated and the 15th result was obtained. In this work, the left and right ventricular stroke work and their sum (Plv, Prv, Ptotal), the left and right ventricular ejection fraction (LVEF, RVEF), the mean Cardiac Output (mCO) and the mean pressure and flow-rate ratio of superior and inferior vena cava (mPsvc\mPivc and mQsvc\mQivc), respectively, were used to describe the hemodynamics of reoperation.

**Results:**

With the change of PVR from one to four times, the values of Plv, Prv, Ptotal, LVEF, and RVEF gradually decreased. The change rate of Plv, Ptotal and LVEF of 1.0VR were the largest in the three kinds of reoperation. The change rate of Prv of 1.5VR′ was larger than that of 2VR, but it was the opposite for their EF change rate. The mCO of 2VR, 1.5VR′, and 1.0VR decreased by 18.53%, 37.58%, and 48.07%, respectively. The mPsvc\mPivc of 1.5VR′ increased from 3.76 to 6.77 and the mQsvc\mQivc decreased from 0.55 to 0.36, while the mPsvc\mPivc and mQsvc\mQivc of 2VR and 1.0VR remained 1 and 0.67, respectively. The peak value of the tricuspid flow-rate (Qti) waveform of 2VR and 1.5VR′ changed from “E peak” to “A peak.”

**Conclusion:**

The numerical results demonstrate the highly reoperation-dependent hemodynamic consequences and their responses to variations in PVR. Comprehensive analysis of EF, mCO and ventricular stroke work indicates that PVR has a greater impact on 1.5VR′ and 1.0VR. Therefore, we suggest that the selection strategy of reoperation should focus on PVR.

## Introduction

The one and a half ventricle repair (1.5VR) is widely applied in the treatment of congenital heart disease with abnormal structure and function of the right ventricle like the Pulmonary Atresia with Intact Ventricular Septum (PA with IVS) (Talwar et al., 2018; Wright et al., 2019), Tetralogy of Fallot (ToF) (Talwar et al., 2018) and Ebstein’s Anomaly (EA) (Malhotra et al., 2018; Talwar et al., 2018; Akkaya et al., 2019). The operation consists of the bidirectional cavopulmonary shunt (BCPS) and the correction of the intracardiac malformation, and aims to make the right ventricle bear only blood flow of the lower body; thus, the sum of the left and right ventricle stroke work (Ptotal) won’t change while the right ventricular volume load is reduced (Barron, 2018). This operation maintains a low right atrial pressure, pulsating pulmonary blood flow and adequate blood oxygen during the short/medium term (Bhattarai et al., 2017; Talwar et al., 2018), which reduces the occurrence of poor prognosis such as the biventricular repair induced right heart failure (Talwar et al., 2018) and the single ventricular repair induced circulatory failure (Sharma et al., 2012; Talwar et al., 2018).

**FIGURE 1:**
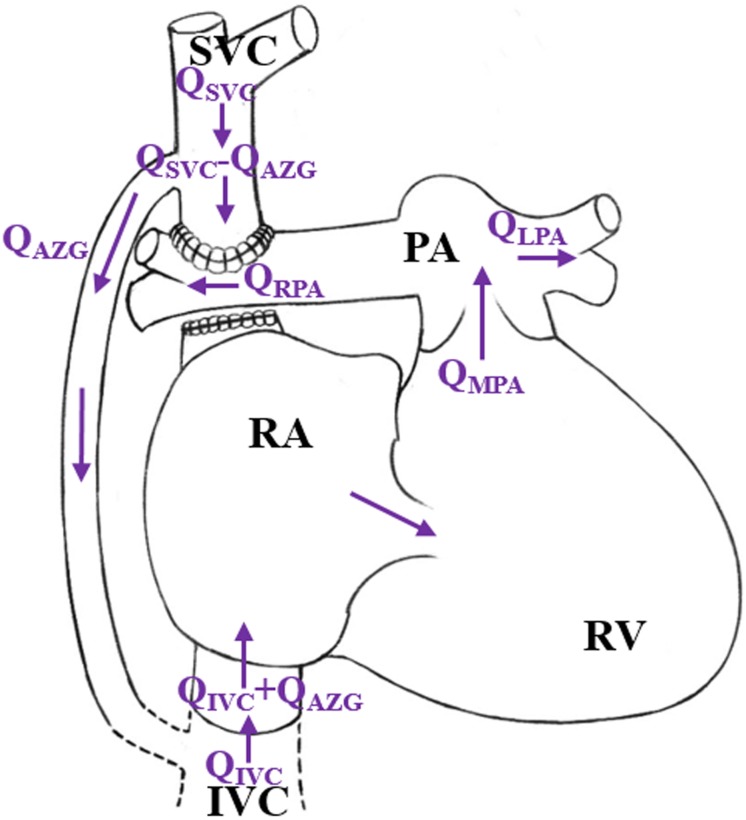
Schematic diagram of “stolen blood” phenomenon of azygos vein. (RA, right atrium; RV, right ventricle; SVC, superior vena cava; IVC, Inferior vena cava; PA, pulmonary artery; Q_*AZG*_, flow-rate of azygos vein; Q_*MPA*_, flow-rate of main pulmonary artery; Q_*LPA*_, flow-rate of left pulmonary artery; Q_*RPA*_, flow-rate of right pulmonary artery; Q_*SVC*_, flow-rate of superior vena cava; Q_*IVC*_, flow-rate of Inferior vena cava).

However, in patients with pulmonary vascular dysplasia or left ventricular dysfunction, complications such as the superior vena cava hypertension and Cardiac Output (CO) reduction are very likely to happen (Liu et al., 2011). When the superior vena cava pressure (Psvc) is higher than the normal value (4–6 mmHg), an adaptive growth of the azygos vein may occur. In the literature, the internal diameter of the azygos vein was positively correlated with Psvc (Preger et al., 1969; Contou et al., 2014), which would lead to a vascular steal phenomenon in the azygos vein, namely the rate of the blood flow from the superior vena cava to the pulmonary artery will decrease, while the blood flow from the azygos vein back to the right ventricle will increase, as shown in Figure 1. This phenomenon causes an increase in the right ventricular preload after 1.5VR. If the Pulmonary Vascular Resistance (PVR) is further increased, irreversible changes will occur in the pulmonary vascular remodeling (Peng et al., 2016), leading to an increase in the right ventricle afterload. Finally, the circulation function after the 1.5VR surgery will deteriorate.

Fortunately, the occurrence of such adverse events can be avoided with reoperation. The clinical reoperation measures include the return biventricular repair (2VR), the ligation of the azygos vein (1.5VR′), the return single ventricular repair (1.0VR), the artificial tonic solution and many others. The selection of the reoperation in clinical practice generally takes into account the morphological parameters and physiological functions of the left and right ventricles (Shimizu et al., 2010). However, there is still a lack of research on the effect of pulmonary vascular development on the hemodynamics after reoperation. Due to the limited number of patients, different conditions of patients and their right ventricles, the effects of pulmonary vascular development on 2VR, 1.5VR′, and 1.0VR cannot be tested through clinical research. For patients with different pulmonary vascular development, it is difficult to design experiments to reproduce the hemodynamics before and after reoperation.

In order to study various hemodynamic problems associated with congenital heart disease (CHD), a wide range of computational models have been established in the literature [i.e., the lumped parameter models (LPM)]. Zhang et al. (2019) developed a zero-dimensional (0D) open-loop model to analyze the hemodynamics in aneurysms. Smith et al. (2004) constructed the smallest human cardiovascular system (OD model) to successfully capture the physiological changes. Ma et al. (2014) constructed an LPM for the blood circulation system of single ventricular patients to observe the recovery of different stenosis degrees of the pulmonary artery after the Gleen surgery. Zhao et al. (2015) used the 0D/3D (three-dimensional) coupling model to study the hemodynamic effects of the anastomoses in modified Blalock-Taussig shunt. Corsini et al. (2018) focused on coronary perfusion and aortic arch hemodynamics using computational multi-domain modeling.

This work aims to develop a simple and effective LPM closed model, which only includes systemic and pulmonary circulation. According to the 1.5VR surgery characteristics, the systemic circulation is divided into upper and lower body blood circulation. Therefore, this work builds on the modeling experience of the former report. We attempted to use the lumped parameter model (LPM) based on the lumped parameter state-variable equation (Di Molfetta et al., 2015; Duanmu et al., 2018) to quantitatively analyze the hemodynamic differences between three reoperation procedures of a given PVR and their sensitivity to PVR. The results will provide guidance to select the strategy for reoperation procedures after 1.5VR failure.

## Materials and Methods

**FIGURE 2:**
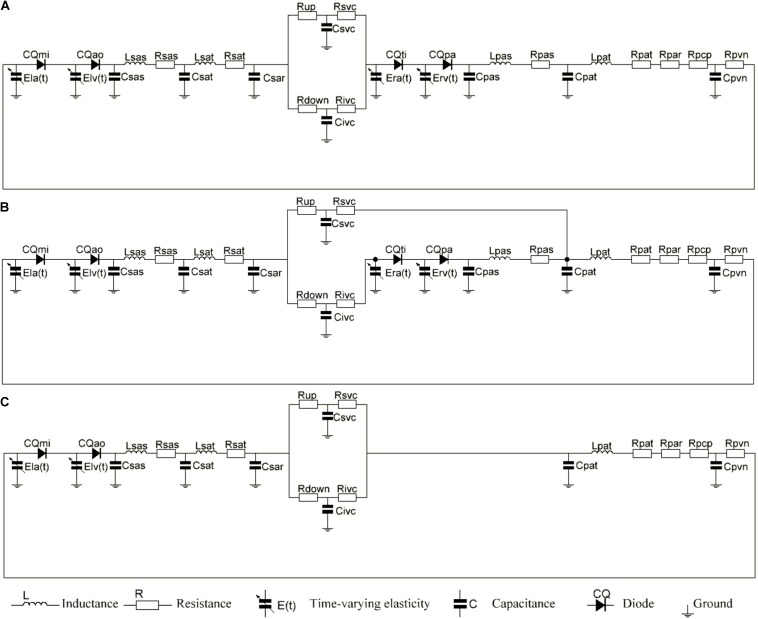
**(A)** The electric equivalent circuit of biventricular repair (2VR). **(B)** The electric equivalent circuit of ligation of azygos vein (1.5VR′). **(C)** The electric equivalent circuit of single ventricular repair (1.0VR). Nomenclature (R, resistance; C, compliance; CQ, flow coefficient; E, elastance; L, inertance). Subscripts (ao, aortic valve; la, left atrium; lv, left ventricle; mi, mitral value; pa, pulmonary aortic valve; par, pulmonary arterioles; pas, pulmonary artery sinus; pat, pulmonary artery; pcp, pulmonary capillary; pvn, pulmonary vein; ra, right atrium; rv, right ventricle; sar, systemic arterioles; sas, systemic aortic sinus; sat, systemic artery; up, upper of body, down lower of body; svc, systemic vena cava; ivc, interior vena cava; ti, tricuspid valve).

The equivalent circuit model that was used to simulate the cardiovascular system is shown in Figure 2. We coupled the Time-varying Elastic Cardiac Cavity Model with the three-element Windkessel vascular model (Korakianitis and Shi, 2006a) to construct a mathematical model of the cardiovascular system after reoperation. The mathematical model consists of three main parts: the heart, the systemic circulation and the pulmonary circulation.

### The Heart

The heart is modeled as a four-chamber pump with a variable elasticity and four heart valves that control the direction of the blood flow.

#### Ventricle

The pressure-volume relationship is used to describe the basic characteristics of the ventricle. Suga et al. (Suga et al., 1973; Suga and Sagawa, 1974) established the idea of a cardiac pump model to simulate the systolic and diastolic functions of the ventricle based on the Frank Starling theory. The ventricular pressure of this model is described as a linear function of the chamber volume and elasticity, and can be mathematically expressed as follows:

(1) $$E\,\left(t\right)=\frac{\text{P}\,\left(\text{t}\right)}{\text{V}\,\left(\text{t}\right)-\text{V}\,0}$$

where E(t) represents the time-varying elasticity, P(t) and V(t) represent the instantaneous ventricular pressure and volume, respectively, and V0 represents the corresponding ventricular volume when the ventricular pressure is 0. The ventricular model in this paper refers to that of Li et al. (2019) and is expressed by equations (2) and (3).

(2) E⁢(t)=(Emax-Emin)⋅En⁢(tn)+Emin

(3) $$\text{En}\,\left(\text{tn}\right)=1.55\cdot \left(\frac{{\left(\frac{\text{tn}}{0.7}\right)}^{1.9}}{1+{\left(\frac{\text{tn}}{0.7}\right)}^{1.9}}\right)\cdot \left(\frac{1}{1+{\left(\frac{\text{tn}}{1.17}\right)}^{21.9}}\right)$$

where En(tn) is the normalized elasticity, also known as the “double hill” function, t_n_ = t/t_max_t_max_ = 0.15t_c_ + 0.2, tc = 60/HR, and refers to a typical cardiac cycle, HR is the heart rate and Emax and Emin are the end-systolic and end-diastolic elasticity of ventricles, respectively. Although the model of the right ventricle is similar to that of the left one, the coefficients of the elastic chamber are different.

#### Atrium

With the generation of the P wave in the electrocardiogram (ECG), the atrial contraction leads to a rapid increase in the ventricular volume and pressure during diastole. This effect is sometimes referred to as the “atrial pulsation,” which accounts for 20–30% of the ventricular filling and reflects the function of the “booster pump” of the atrium (Korakianitis and Shi, 2006a). The modeling process of the atrium is similar to that of the ventricle. According to the reference (Korakianitis and Shi, 2006b), this essay describes the dynamic changes of the atrium with the parameter values and activation function. Taking the left atrium as an example, the equations of the elastic time-varying function and activation function are shown in Eq. 4 and Eq. 5, respectively.

(4) Ela=(Elamax-Elamin)⋅Enla+Elamin

(5) Enla={ 0                            0≤t<T⁢p⁢w⁢b 1-cos⁡(t-TpwbTpww⋅ 2⁢π)  Tpwb≤t<T⁢p⁢w⁢b+T⁢p⁢w⁢w 0      T⁢p⁢w⁢b+T⁢p⁢w⁢w≤t<t⁢c

where Tpwb = 0.92^∗^tc is the start time of the P wave in ECG, Tpww = 0.08^∗^tc represents its duration time and Elamax and Elamin show the elasticity of the end-systolic and end-diastolic of the left atrium, respectively. The modeling equation of the right atrium is the same as that of the left atrium, but the parameter values are different.

#### Valve

A diode is used to control the one-way flow of blood in the LPM. The basic pressure-flow relationship of the valve is described using the “hole” model in fluid mechanics (Korakianitis and Shi, 2006a). Taking the mitral valve as an example, its mathematical expression is shown in Eq. 6 and Eq. 7.

(6) $$\text{Qmi}=\left\{ \begin{array}{c} C\,Q\,m\,i\cdot A\,R\,m\,i\cdot \sqrt{\text{Pla}-\text{Plv}}\,P\,l\,a>P\,l\,v\\ C\,Q\,m\,i\cdot A\,R\,m\,i\cdot \sqrt{\text{Plv}-\text{Pla}}\,P\,l\,v>P\,l\,a \end{array}\right.$$

(7) ARmi={ 1,P⁢l⁢a>P⁢l⁢v 0,P⁢l⁢v>P⁢l⁢a

ARmi is the ratio of the valve open area to the maximum area. In this work, the movement process of the valve was ignored, and the movement of the valve was simplified into two states of being fully opened (1) and completely closed (0). Whether the valve is open or not depends on the pressure at both ends of the valve. Finally, CQmi is the valve flow coefficient, which is related to the orifice area when the valve opens.

### Blood Circulation Circuit

The systemic circulation was divided into systemic aortic sinus, systemic artery, systemic arterioles and systemic vein. In order to simulate the hemodynamics of the reoperation after 1.5VR failure, systemic arterioles and systemic vein were each divided into two parts: the upper loop and the lower loop (Shimizu et al., 2010). The pulmonary circulation was mainly composed of pulmonary artery sinus, pulmonary artery, pulmonary arterioles, pulmonary capillary and pulmonary vein. Constructing the LPM of the vessel segment needs to focus on the local flow condition (Corsini et al., 2014, 2018). Based on the electrical-fluid parameters, the circuit model of the resistance (R), capacitance (C) and inductance (L) corresponding to the viscosity, elastic coefficient and inertia of the fluid model were applied to mimic the blood flow impedance, blood flow compliance and blood inertia, respectively. Among them, the aortic sinus and artery have better elasticity, and their blood flow is pulsatile. Therefore, the effects of R, C, and L should be considered when modeling these vascular segments. Additionally, the blood flow in the arterioles was relatively smooth, and only the effects of C and R were considered. Moreover, only the R effect needs to be considered for pulmonary capillaries. Otherwise, the main function of the veins is to collect and store blood, so the effects of C and R were considered in venous modeling. The final circuit model is shown in Figure 2. The pressure on each C (Pcc) is defined in Eq. 8.

(8) $$\frac{\text{dPcc}}{\text{dt}}=\frac{\sum \text{Qin}\,\left(\text{t}\right)-\sum \text{Qout}\,\left(\text{t}\right)}{\text{C}}$$

If the effect of L is to be considered, then the flow rate of each part is defined as Eq. 9. Otherwise, it is defined as Eq. 10.

(9) $$\frac{\text{dQin}\,\left(\text{t}\right)}{\text{dt}}=\frac{\text{Pin}\,\left(\text{t}\right)-\text{Pout}\,\left(\text{t}\right)-\text{Qin}\,\left(\text{t}\right)*R}{\text{L}}$$

(10) $$\text{Qin}\,\left(\text{t}\right)=\frac{\text{Pin}\,\left(\text{t}\right)-\text{Pout}\,\left(\text{t}\right)}{\text{R}}$$

**FIGURE 3:**
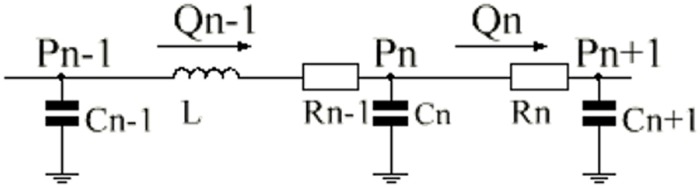
Single-compartment circuit representation. P, pressure; R, resistance; C, compliance; L, inertance; Q, flow-rate, n–1, n and n+1, compartment indexes.

See Figure 3 illustrates the definition of subscripts in these equations.

### Determining the LPM Parameters

The values of the physiological parameters are generally difficult to measure and vary among individuals. The parameter values in the LPM of reoperation constructed in this work are widely referenced from the literature to select appropriate values. However, in order to simulate different physiological realities, the parameters usually need to be adjusted. It is well known from the literature that the blood flow rate of the descending aorta accounts for 63.8% of CO, and the compliance of the inferior vena cava accounts for 66.6% of the total compliance (Shimizu et al., 2010). Therefore, in this essay, the compliance of the inferior vena cava was set as 0.6 times of the total compliance, and the ratio of the circulating flow of upper body to that of the lower body was 2/3 by adjusting the values of Rup, Rsvc, Rdown, and Rivc. Based on references (Burkhoff and Tyberg, 1993; Heldt et al., 2002), the parameters were adjusted to make the simulation results of 2VR conform to the clinically measured physiological data. The model parameters in this paper are shown in Table 1. MATLAB was used to simulate the effect of PVR on the hemodynamics of the reoperation after 1.5VR failure, in which the value of PVR, defined as the sum of Rpar and Rpcp, was successively increased by 1, 2, 3, and 4 times, while other parameters remained unchanged.

**TABLE 1:** Parameters used in model.

Heart rate (HR), beats/min			75			
Duration of cardiac cycle (tc), s			0.8			
Beginning of P wave in ECG (Tpwb), s			0.92*tc			
Duration of P wave in ECG (Tpww), s			0.08*tc			

			**LV**	**RV**	**LA**	**RA**

End-systolic elastance (Ees), mmHg/mL			2.5	1.15	0.2	0.25
End-diastolic elastance (Eed), mmHg/mL			0.05	0.07	0.15	0.15
Unstressed volume (V0), mL			5	10	0	0

			**Mitral**	**Aortic**	**Tricuspid**	**Pulmonary aortic**

Valve flow coefficient (CQ), ml/(s⋅mmHg^0.5^)			400	350	400	350

	**Resistance (R), mmHg⋅s/mL**		**Compliance (C), mL/mmHg**		**Inertance (L), mmHg⋅s^2^/mL**	

Systemic circulation	Rsas	0.003	Csas	0.08	Lsas	1.86E-05
	Rsat	0.05	Csat	0.7	Lsat	0.0017
	Rup	2.325	Csar	0.5		
	Rsvc	0.0375	Csvc	8.2		
	Rivc	0.025	Civc	12.3		
	Rdown	1.55				
Pulmonary circulation	Rpas	0.002	Cpas	0.18	Lpas	1.56E-05
	Rpat	0.01	Cpat	4.75	Lpat	0.0017
	Rpar	0.08	Cpvn	20.5		
	Rpcp	0.045				
	Rpvn	0.015				

### Hemodynamic Parameters of Reoperation

The parameters of Plv, Prv, Ptotal, LVEF, RVEF, mPsvc\mPivc, and mQsvc\mQivc were used to describe the hemodynamics after reoperation in this essay. The ventricular work is calculated using MATLAB to integrate the area surrounding the ventricular P – V curve. EF can be calculated by dividing the ventricular stroke volume (SV) by the ventricular end-diastolic volume (Ved), while SV is equal to Ved and the ventricular end-systolic volume (Ves). The influence of PVR on the ventricular work and the ventricular EF is represented by the change rate of the ventricular work and ventricular EF, respectively. The changing rates of LVEF, RVEF, and mCO are comprehensively analyzed and can reflect the degree of impact of PVR impact on the reoperation.

## Results

It can be seen from Table 2 that the hemodynamic parameters of 2VR at each position simulated under the system parameters listed in Table 1 are consistent with the clinically measured physiological ranges.

**TABLE 2:** Simulation results and normal physiological range data.

Parameter	Simulation results	Physiological range
Left ventricular end-systolic pressure (Plves), mmHg	116.1	90–140
Left ventricular end-diastolic pressure (Plved), mmHg	4.206	0–10
Left atrium end-systolic pressure (Plaes), mmHg	7.979	5–10
Left atrium end-diastolic pressure (Plaed), mmHg	4.681	–2–5
Systemic aortic sinus end-systolic pressure (Psas_es), mmHg	116	90–140
Systemic aortic sinus end-diastolic pressure (Psas_ed), mmHg	74.19	60–90
Right ventricular end-systolic pressure (Prves), mmHg	31.98	18–35
Right ventricular end-diastolic pressure (Prved), mmHg	4.195	0–10
Right atrium end-systolic pressure (Praes), mmHg	7.727	5–10
Right atrium end-diastolic pressure (Praed), mmHg	4.649	–2–5
Pulmonary artery sinus end-systolic pressure (Ppas_es), mmHg	31.7	18–35
Pulmonary artery sinus end-diastolic pressure (Ppas_ed), mmHg	9.617	6–15
Stroke volume (SV), mL	72.8	60–80
Mean cardiac output (mCO), L/min	5.46	3.5–5.5

**FIGURE 4:**
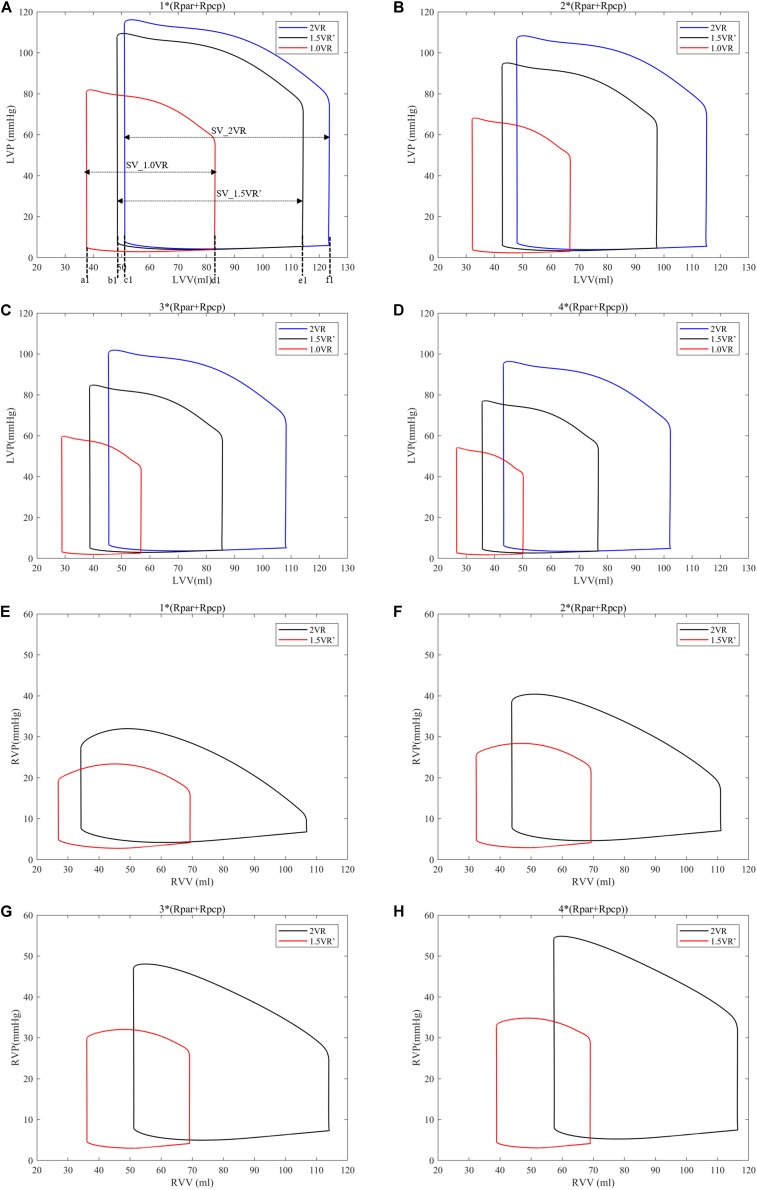
**(A)** Left ventricular P-V curve for three procedures when PVR is 1*(Rpar + Rpcp). **(B)** Left ventricular P-V curve for three procedures when PVR is 2*(Rpar + Rpcp). **(C)** Left ventricular P-V curve for three procedures when PVR is 3*(Rpar + Rpcp). **(D)** Left ventricular P-V curve for three procedures when PVR is 4*(Rpar + Rpcp). **(E)** Right ventricular P-V curve for three procedures when PVR is 1*(Rpar + Rpcp). **(F)** Right ventricular P-V curve for three procedures when PVR is 2*(Rpar + Rpcp). **(G)** Right ventricular P-V curve for three procedures when PVR is 3*(Rpar + Rpcp). **(H)** right ventricular P-V curve for three procedures when PVR is 4*(Rpar + Rpcp). a1, b1, and c1, left ventricular end-systolic volume as indicated. d1, e1, and f1, left ventricular end-diastole volume as indicated. SV, stroke volume. (Rpar, resistance of pulmonary arterioles; Rpcp, resistance of pulmonary capillary; LVP, left ventricular pressure; LVV, left ventricular volume; RVP, right ventricular pressure; RVV, right ventricular volume; P-V, pressure-volume).

**FIGURE 5:**
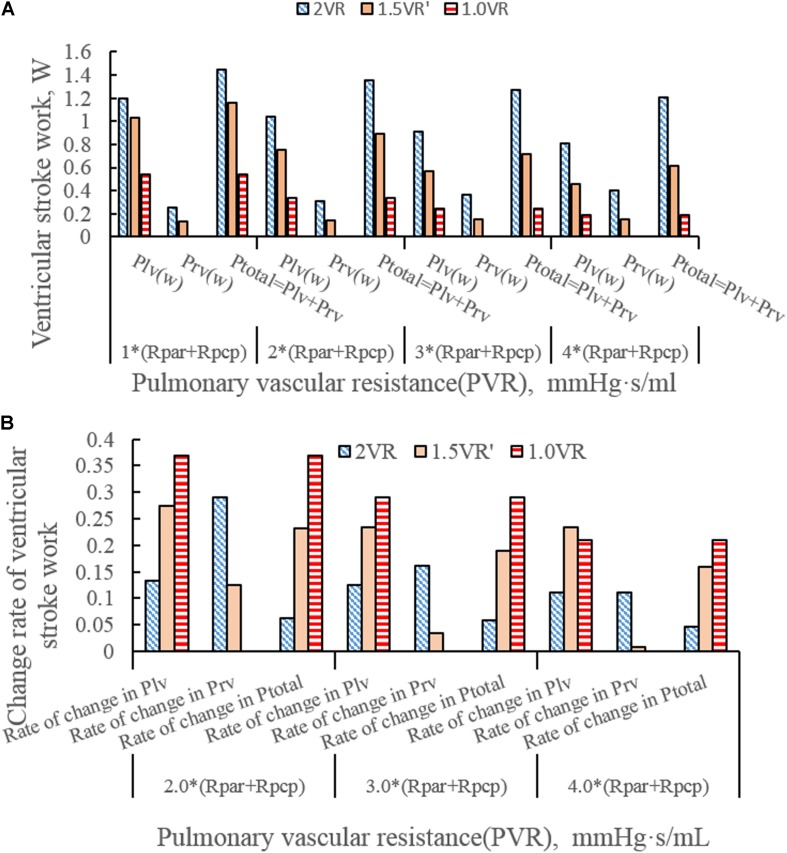
**(A)**Ventricular stroke work in three procedures under different PVR. **(B)** Change rate of ventricular stroke work in three procedures under different PVR. (Plv, left ventricular stroke work; Prv, right ventricular stroke work; Ptotal, The sum of left ventricular and right ventricular of stroking work; Rpar, resistance of pulmonary arterioles; Rpcp, resistance of pulmonary capillary).

**FIGURE 6:**
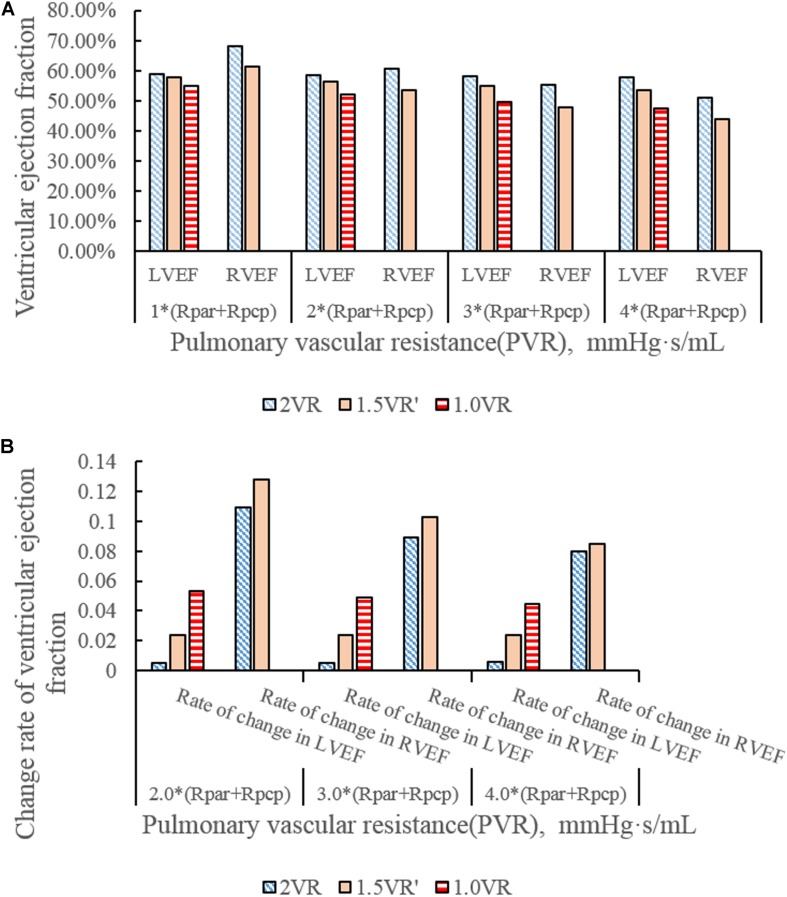
**(A)**ventricular ejection fraction in three procedures under different PVR. **(B)** Change rate of ventricular ejection fraction in three procedures under different PVR.

Figures 4A–H show the impact of PVR on the stroke work of the left ventricle and right ventricle after each reoperation procedure; the equivalent quantitative results are shown in Figure 5A. Within the range from 1 time to 4 times of PVR, in 2VR, Plv decreased from 1.2 W to 0.81 W, Prv increased from 0.25 W to 0.4 W and Ptotal decreased from 1.45 W to 1.21 W. In 1.5VR′, Plv decreased from 1.03 W to 0.46 W, Prv increased from 0.13 W to 0.15 W and Ptotal decreased from 1.16 W to 0.61 W. In 1.0VR, Plv and Ptotal both decreased from 0.54 W to 0.19 W. Within the range from 1 to 4 times of PVR, the rate of change of Plv and Ptotal of 1.0VR was obviously greater than that of other reoperation producers, while the rate of change of Prv of 2VR was apparently greater than that of 1.5VR′ (Figure 5B). The ventricular EF is closely related to SV, end systolic volume (Vlves) and end diastolic volume (Vlved). Figure 6A shows that with the increase of PVR, LVEF decreased from 58.80% to 57.86% and RVEF decreased from 68.09% to 50.87% in 2VR. In 1.5VR′, LVEF decreased from 57.73% to 53.66%, and RVEF decreased from 61.31% to 43.86%. In 1.0VR, LVEF decreased from 54.93% to 47.28%. As the PVR increased from 1 to 4 times, the changing rate of LVEF in 1.0VR was obviously greater than that of other reoperation producers, while the changing rate of RVEF in 1.5VR′ was apparently greater than that of 2VR. Detailed data can be obtained from Figure 6B.

**FIGURE 7:**
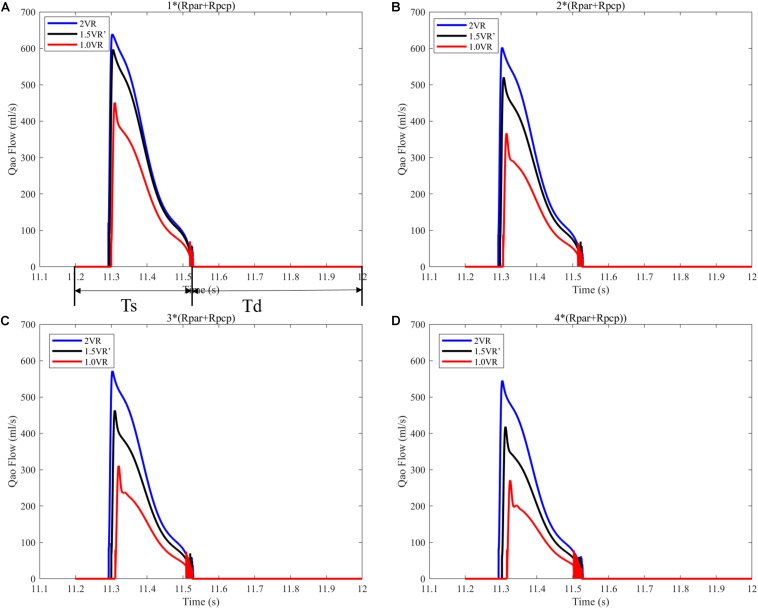
**(A)** When the PVR is 1*(Rpar + Rpcp), the cardiac output of the three procedures. **(B)** When the PVR is 2*(Rpar + Rpcp), the cardiac output of the three procedures. **(C)** When the PVR is 3*(Rpar + Rpcp), the cardiac output of the three procedures. **(D)** When the PVR is 4*(Rpar + Rpcp), the cardiac output of the three procedures. Ts is the duration of systolic time of left ventricle in a typical cardiac cycle. Td is the duration of diastole time of left ventricle in a typical cardiac cycle. (Qao, cardiac output; PVR, pulmonary vascular resistance; Rpar, resistance of pulmonary arterioles; Rpcp, resistance of pulmonary capillary).

**FIGURE 8:**
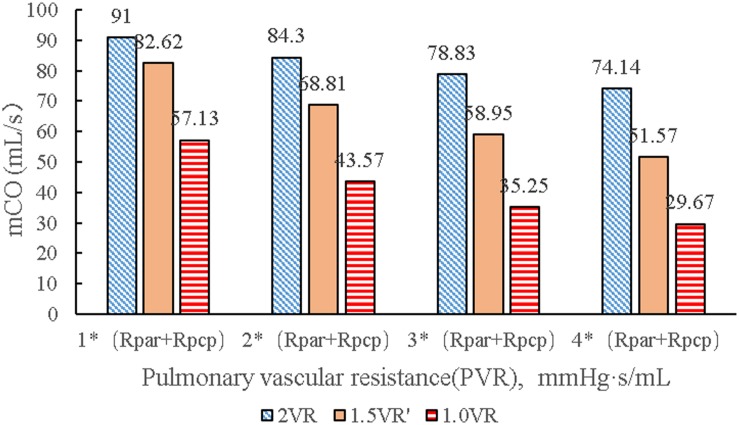
mCO in three procedures under different PVR. (mCO, mean cardiac output; Rpar, resistance of pulmonary arterioles; Rpcp, resistance of pulmonary capillary).

It can be seen from Figures 7A–D that the CO difference among the three kinds of surgical operations was obvious, and the CO of each kind gradually decreased with the increase of PVR. During the systolic time of the left ventricle (Ts), when PVR gradually increased, mCO of 2VR, 1.5VR′, and 1.0VR decreased by 18.53%, 37.58%, and 48.07%, respectively (Figure 8).

**FIGURE 9:**
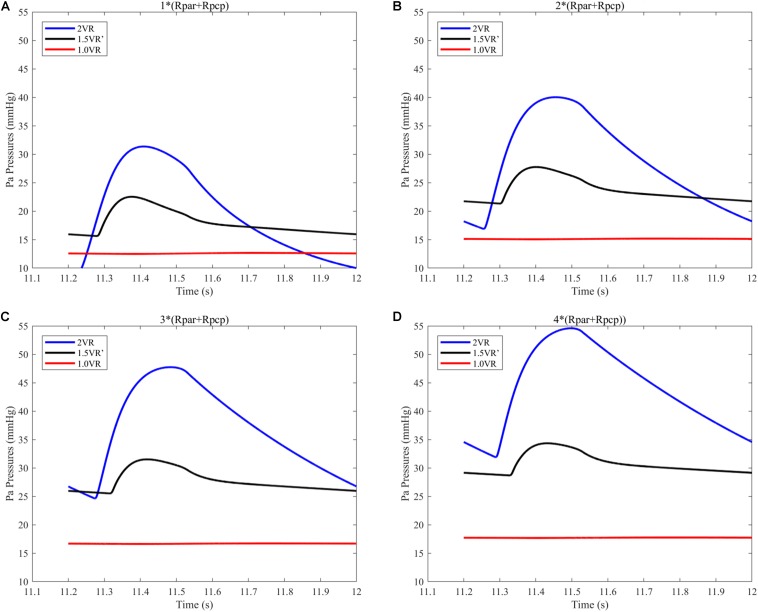
**(A)** When the PVR is 1*(Rpar + Rpcp), Pa pressure of the three procedures. **(B)** When the PVR is 2*(Rpar + Rpcp), Pa pressure of the three procedures. **(C)** When the PVR is 3*(Rpar + Rpcp), Pa pressure of the three procedures. **(D)** When the PVR is 4*(Rpar + Rpcp), Pa pressure of the three procedures. (Pa, pulmonary artery; Rpar, resistance of pulmonary arterioles; Rpcp, resistance of pulmonary capillary).

**FIGURE 10:**
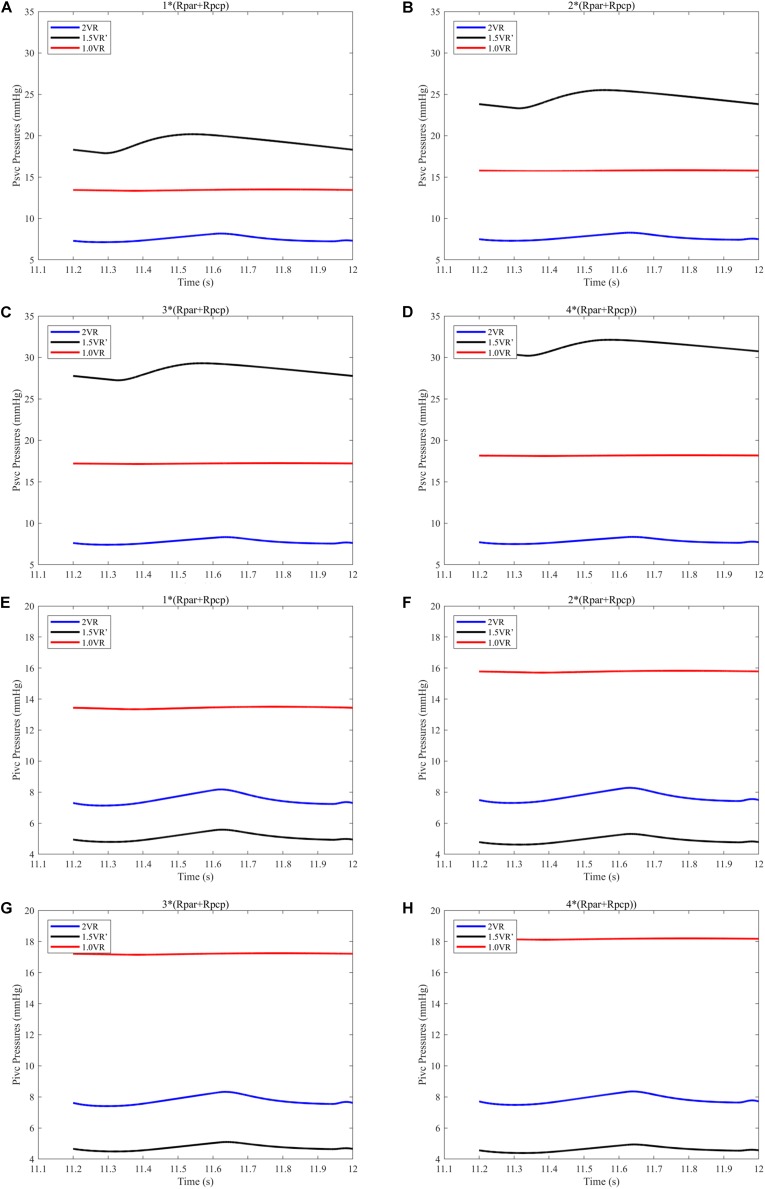
**(A)** When the PVR is 1*(Rpar + Rpcp), Psvc of the three procedures. **(B)** When the PVR is 2*(Rpar + Rpcp), Psvc of the three procedures. **(C)** When the PVR is 3*(Rpar + Rpcp), Psvc of the three procedures. **(D)** When the PVR is 4*(Rpar + Rpcp), Psvc of the three procedures. **(E)** When the PVR is 1*(Rpar + Rpcp), Pivc of the three procedures. **(F)** When the PVR is 2*(Rpar + Rpcp), Pivc of the three procedures. **(G)** When the PVR is 3*(Rpar + Rpcp), Pivc of the three procedures. **(H)** When the PVR is 4*(Rpar + Rpcp), Pivc of the three procedures. (Psvc, superior vena cava pressure; Pivc, inferior vena cava pressure; Rpar, resistance of pulmonary arterioles; Rpcp, resistance of pulmonary capillary).

**FIGURE 11:**
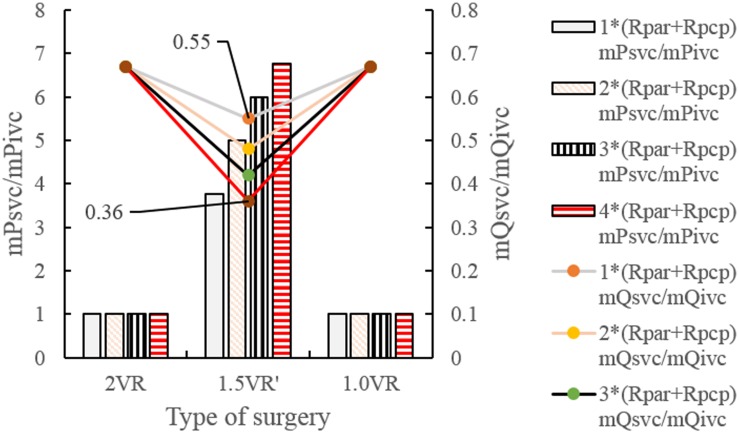
mPsvc/mPivc and mQsvc/mQivc in three procedures under different PVR. (mPsvc mean superior vena cava pressure, mPivc mean inferior vena cava pressure, mQsvc mean flow-rate superior vena cava, mQivc mean flow-rate of inferior vena cava pressure, Rpar resistance of pulmonary arterioles, Rpcp resistance of pulmonary capillary).

The results for Pa, Psvc and Pivc are shown in Figures 9A–D and Figures 10A–H, respectively. In 1.0VR, their wave was without pulsation when PVR increased. Regardless of the condition of PVR, Psvc was always greater than Pivc, and Pa was equal to Psvc in 1.5VR′ in a cardiac cycle. The influence of PVR on mPsvc/mPivc is shown in Figure 11. The ratio mPsvc\mPivc of 2VR and 1.0VR was 1, but it increased from 3.76 to 6.76 when PVR increased in 1.5VR′.

**FIGURE 12:**
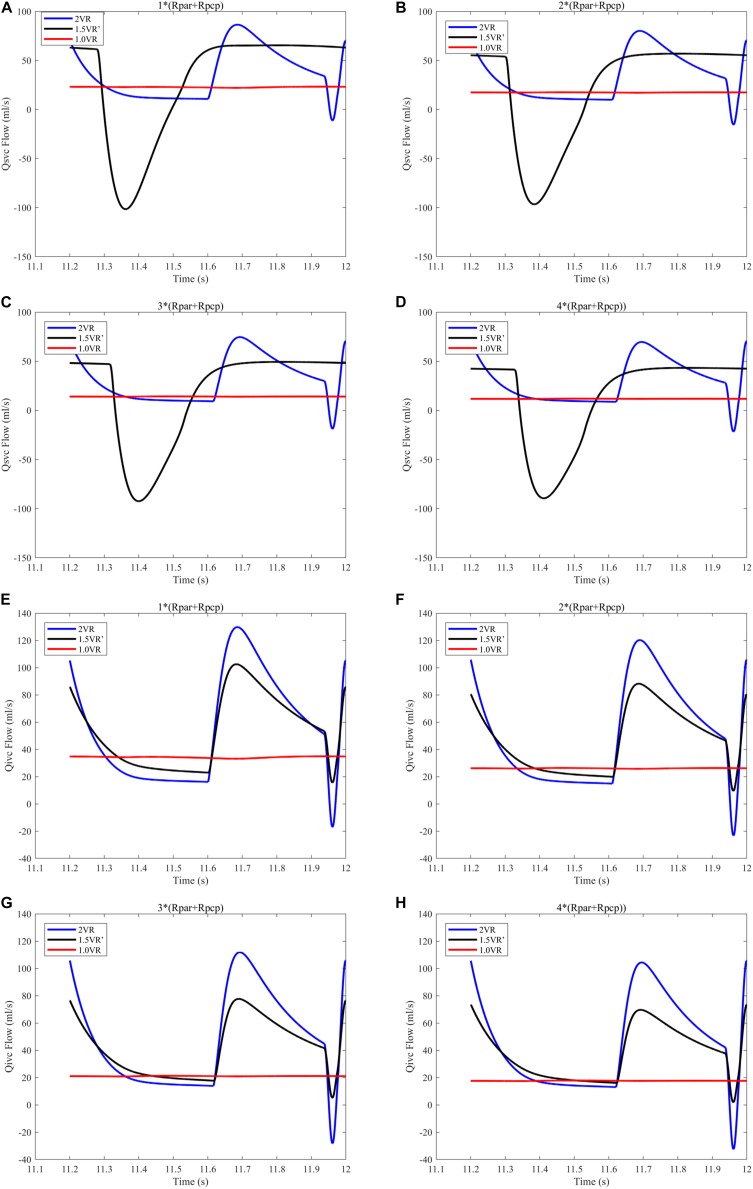
**(A)** When the PVR is 1*(Rpar + Rpcp), Qsvc of the three procedures. **(B)** When the PVR is 2*(Rpar + Rpcp), Qsvc of the three procedures. **(C)** When the PVR is 3*(Rpar + Rpcp), Qsvc of the three procedures. **(D)** When the PVR is 4*(Rpar + Rpcp), Qsvc of the three procedures. **(E)** When the PVR is 1*(Rpar + Rpcp), Qivc of the three procedures. **(F)** When the PVR is 2*(Rpar + Rpcp), Qivc of the three procedures. **(G)** When the PVR is 3*(Rpar + Rpcp), Qivc of the three procedures. **(H)** When the PVR is 4*(Rpar + Rpcp), Qivc of the three procedures. (Qsvc, flow-rate superior vena cava; Qivc, flow-rate of inferior vena cava; Rpar, resistance of pulmonary arterioles; Rpcp, resistance of pulmonary capillary).

**FIGURE 13:**
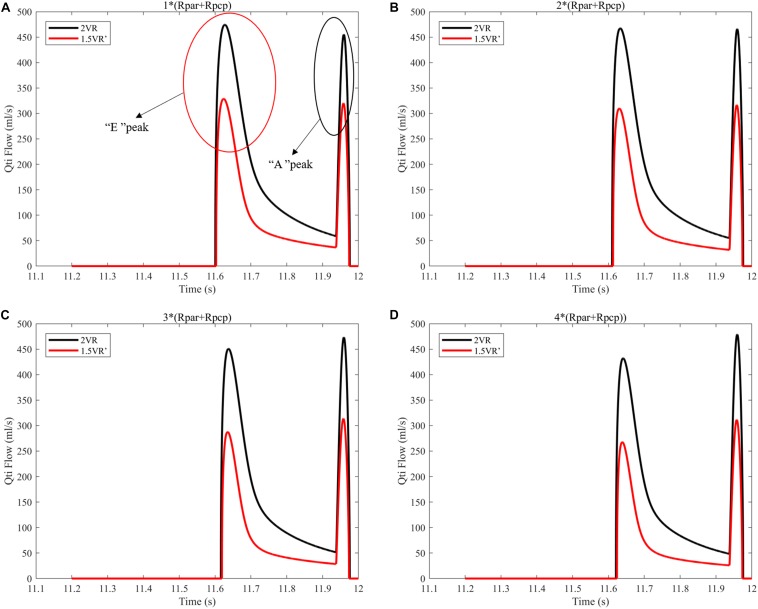
**(A)** When the PVR is 1*(Rpar + Rpcp), Qti of different procedures. **(B)** When the PVR is 2*(Rpar + Rpcp), Qti of different procedures. **(C)** When the PVR is 3*(Rpar + Rpcp), Qti of different procedures. **(D)** When the PVR is 4*(Rpar + Rpcp), Qti of different procedures. (Qti, flow-rate of right atrium; Rpar, resistance of pulmonary arterioles; Rpcp, resistance of pulmonary capillary).

Figures 12A–H and Figures 13A–D show the impact of PVR on Qsvc, Qivc and Qti, respectively. Qsvc of 1.5VR′ have a negative value in the period of 11.2 s ∼ 11.5 s (Ts), while Qsvc of 2VR have a negative value in the period of 11.9 s ∼ 12.0 s (Td). In 1.0VR, the wave of Qsvc and Qivc was without pulsation when PVR increased. With the increase of PVR, the peak of waveform of Qti changed from “E peak” to “A peak” in 2VR and 1.5VR′. The change in the pressure is the cause of the flow-rate redistribution. Therefore, as PVR increased from 1 to 4 times, mQsvc\mQivc of 1.5VR′ decreased from 0.55 to 0.36, while it was 0.67 in both 2VR and 1.0VR (Figure 11).

## Discussion

1.5VR has been employed as a useful surgery in patients with CHD. The postoperative results of the surgery are highly patient-specific. In this paper, we have established three general LPM models, representing reoperation procedures after the failure of 1.5VR. They are closed-loop models that only consider the systemic and pulmonary circulation, with the exclusion of the local fine renal and liver circulation. The purpose of developing general LPM models is to mimic the postoperative hemodynamics of reoperation on specific PVR. These models are simple and effective. In addition, based on the models of this paper, we can adjust the lumped parameters of each vascular segment such as R, C, L, and others, so that clinical phenomena such as the azygos vein growth and the results of surgical procedures can be reproduced. The study parameters include Plv, Prv, Ptotal, CO, Qti, Pa, Psvc, Pivc, Qsvc, and Qivc.

The ventricular stroke work can directly reflect the function of the ventricle. With the increase of PVR, The value of Prv of 2VR and 1.5VR′ gradually increased. This phenomenon is in accordance with the qualitative results inferred from general cardiovascular physiology. Furthermore, the Plv/Prv of 1.5VR′ exceeded that of 2VR, which is consistent with the purpose of the 1.5VR surgery (Talwar et al., 2018). Considering the changing rates in the ventricular stroke work of the three surgical procedures, Plv and Ptotal of 1.0VR are more sensitive to variations in PVR, while Prv of 2VR is more sensitive to changes in PVR. The ventricular EF also has a higher correlation with the ventricular function. Under different PVR conditions, the LVEF and RVEF of the three surgeries in this paper were all about 50%. This result indicates a better ventricular function under the given PVR (Surkova et al., 2019). As PVR increases, the afterload of the right ventricle increases and EF decreases. The results shown in Figure 6 are consistent with trends that are qualitatively inferred from general cardiovascular physiology. As seen from the factor of the changing rate of LVEF and RVEF, RVEF is more sensitive to the change of PVR in 2VR and 1.5VR′.

Under different PVR conditions, LVEF and RVEF of the three surgeries stay around 50% in this work, while the mCO values of 1.5VR′ and 1.0VR are obviously decreased. In other words, the filling of the left ventricle decreases (Verhoeff and Mitchell, 2017), which is consistent with the clinical practice. In the clinical situation, the patient with chronic thromboembolic pulmonary hypertension will have an increase in PVR, and the function of the right heart is particularly important (Ruigrok et al., 2019); thus, maintaining a normal CO (3.5 L/min–5.5 L/min) is very important for normal blood circulation of the human body.

In turn, changes in the ventricular function affect changes in the pressure and flow in various parts of the body. The waves of Pa, Psvc, Pivc, Qsvc, and Qivc are of no pulsation in 1.0VR, which is determined by the surgical characteristics of 1.0VR (Jarvis et al., 2016) and is consistent with the clinical practice. With the increase of PVR, Pa of all three surgical methods increases, resulting in different degrees of pulmonary hypertension (Palazzini et al., 2017). Psvc in 1.5VR′ is greater than Pivc due to the characteristics of 1.5VR′ (Bhattarai et al., 2017). mPsvc\mPivc of 1.5VR′ is the largest among the three kinds of procedures, that is because in 1.5VR′, the superior vena cava anastomoses to the pulmonary artery, and Psvc increases due to the influence of Pa (Bhattarai et al., 2017). During the period Ts, the right ventricle is in systole, resulting in Ppa greater than Psvc in 1.5VR′, then more blood would flow from the pulmonary artery to the superior vena cava; thus, the waveform of Qsvc is negative during Ts. However, the superior vena cava in 2VR is connected to the right atrium, and the negative waveform of Qivc during the period of Td is due to the active contraction of the right atrium. Moreover, mPsvc\mPivc is the reason for the change of mQvc\mQivc which has the smallest value in 1.5VR′, this will lead to a significant decrease in blood perfusion in the upper body.

In addition, Qti is closely related to the ventricular function. The “E peak” and “A peak” of Qti waveform are generated by the active systole of the right ventricle and right atrium, respectively, so the time of occurrence of the peak is different. With the increase of PVR, the waveform peak of Qti changes from “E peak” to “A peak,” which indicates that PVR can affect the right ventricular function to some extent. In reference (Murayama et al., 2019), the ratio of velocity between “E peak” and “A peak” is used to quantify the ventricular diastolic function.

## Limitations

A major limitation of this study is related to the model we established. The model constructed in this essay is relatively simple and does not take into account the factors of interventricular interaction, valve movement and coronary circulation of the great clinical value, although it effectively captures physiological changes. Some scholars have used computational models to verify the importance of interventricular interaction (Korakianitis and Shi, 2006a; Lin et al., 2019) and valve movement (Korakianitis and Shi, 2006a). However, 1.5VR that is generally selected for patients based on their cardiac appearance is characterized by the right ventricle being much smaller than the left one, so the interventricular interaction can be ignored. The other limitation is the lumped parameter, which is retrieved from the value of normal people in literature. However, the parameter for CHD is different from that of the normal people. Due to the limited cases of patients with reoperation after 1.5VR failure, it is difficult to obtain actual clinical data. It is well-known that the lumped parameters determined by the normal people are somewhat different from those of a patient, but the interaction between the peripheral tissues of the human body is almost the same, so the lumped parameters determined by a normal person can be used for patients. In addition, only PVR changes are allowed in the simulation process, while other parameters remain unchanged. It has been reported that PVR changes are caused by pulmonary vascular histology, and the resulting pulmonary hypertension will also affect the physiological properties of other parts, such as decreased pulmonary vascular compliance, etc. (Dragu et al., 2015). In future studies, we can analyze the preoperative data of more patients to solve this problem.

## Conclusion

By constructing the LPM of the reoperation after 1.5VR failure, we investigated the three kinds of surgical procedures, including the 2VR, 1.5VR′, and 1.0VR. Through MATLAB simulation analysis, the hemodynamic differences among the three kinds of surgical procedures are quantitatively revealed at different PVR. In this paper, we mainly focus on some values which are the ventricular stroke work, EF, Cardiac Output and pressure and flow rate of the superior and inferior vena cava, which can directly or indirectly reflect the function of the heart. Using these values, we can describe the hemodynamics of reoperation after 1.5VR failure under different PVR. The ventricular stroke work, EF and mCO of the changing rate are important parameters that can reflect the sensitivity of different surgical procedures to PVR. Comprehensive analyses of the above-mentioned parameters show that PVR has a greater impact on 1.5VR′ and 1.0VR. Therefore, the selection of reoperation strategies should be mainly based on the development of pulmonary vasculature. To be noted, the definite outcomes of related procedures need to be verified by further experiments or clinic data.

## Data Availability Statement

All datasets generated for this study are included in the article/supplementary material.

## Author Contributions

YF designed and simulated the LPM of virtual surgery, performed the data analysis, and prepared the initial draft manuscript under the instruction of AQ. YY and XF provided the support of clinical knowledge. AQ subsequently revised the manuscript. All authors approved the final submitted version.

## Conflict of Interest

The authors declare that the research was conducted in the absence of any commercial or financial relationships that could be construed as a potential conflict of interest.
